# Data-Driven Comorbidity Phenotypes and In-Hospital Outcomes Among Patients With Nonvariceal Upper Gastrointestinal Bleeding: Formative Retrospective Cohort Study

**DOI:** 10.2196/108793

**Published:** 2026-10-19

**Authors:** Fangning Wang, Yuhang Zhou, Qin Zhang, Yuan Liu, Yanli Zhang, Shiyu Du

**Affiliations:** 1 Department of Gastroenterology, China-Japan Friendship Hospital, Chinese Academy of Medical Sciences & Peking Union Medical College, Yinghuayuan East Street, Beijing, Chaoyang District, 100029, China, +86 13911531937; 2 Department of Gastroenterology, China-Japan Friendship Hospital, Beijing, Chaoyang DistrictChina; 3 Beijing University of Chinese Medicine, Beijing, China

**Keywords:** nonvariceal upper gastrointestinal bleeding, comorbidity phenotype, hospital discharge records, unsupervised machine learning, risk assessment, health care use

## Abstract

**Background:**

Existing risk scores for nonvariceal upper gastrointestinal bleeding (NVUGIB) may not adequately represent the heterogeneity of coexisting chronic conditions. Routinely collected hospital discharge records may enable data-driven characterization of clinically meaningful comorbidity profiles.

**Objective:**

This formative study aimed to identify reproducible comorbidity profiles among patients hospitalized with NVUGIB and examine their associations with in-hospital outcomes and health care use.

**Methods:**

We conducted a retrospective cohort study of 3193 adults hospitalized with NVUGIB at a tertiary hospital in China between 2015 and 2025. Of 55 prespecified chronic conditions, 33 with a prevalence of at least 1% were included as binary clustering variables. Patients were randomly divided into an 80% training subset and a 20% held-out validation subset. Comorbidity profiles were identified in the training subset using a self-organizing map and Ward hierarchical clustering and then assigned to the validation subset using the fixed clustering solution. Profile reproducibility was evaluated by comparing condition prevalence vectors between subsets. Multivariable regression models assessed associations with severe in-hospital outcomes, intensive care unit admission, in-hospital death, length of stay, and hospital costs.

**Results:**

At least 1 selected comorbidity was recorded in 89.7% (2864/3193) of patients. Six profiles were identified: low-burden heterogeneous (cluster 1), non–upper gastrointestinal neoplastic-metastatic (cluster 2), circulatory-renal (cluster 3), cardiometabolic-renal (cluster 4), atherosclerotic-vascular (cluster 5), and rheumatologic-neurological (cluster 6). Profile composition was broadly reproducible between the training and validation subsets (weighted mean Spearman correlation=0.891, SD 0.04; *P*=.001). The circulatory-renal profile had the strongest associations with severe in-hospital outcomes (adjusted odds ratio [aOR] 8.57, 95% CI 5.39-14.01), intensive care unit admission (aOR 10.38, 95% CI 4.87-25.76), and in-hospital death (aOR 4.66, 95% CI 1.97-13.75). The rheumatologic-neurological and cardiometabolic-renal profiles were also associated with higher odds of these outcomes. All 6 profiles were associated with longer hospital stays and higher hospital costs.

**Conclusions:**

Using routinely collected hospital discharge records, we identified clinically interpretable comorbidity profiles among patients with NVUGIB. The profiles were broadly reproducible within the study population and showed different associations with clinical outcomes and resource use. Profile-based information may complement established risk assessment and could be examined further as an electronic health record–based approach to clinical management and resource planning.

## Introduction

Nonvariceal upper gastrointestinal bleeding (NVUGIB) is a major medical emergency associated with substantial clinical and economic burdens. While advances in endoscopic therapy and pharmacological management have improved care, a significant proportion of patients still experience adverse outcomes requiring intensive intervention. Current clinical guidelines emphasize early risk stratification to guide triage. Validated tools such as the Glasgow-Blatchford Score (GBS) are widely used to identify low-risk patients suitable for outpatient management [1-3].

However, existing risk scores primarily focus on acute physiological derangements and laboratory findings. These models often provide a narrow assessment of comorbid conditions, typically treating them as isolated binary variables or simple cumulative counts [1,3]. Patients hospitalized with NVUGIB commonly have one or more coexisting chronic conditions that may reduce physiological reserve, influence treatment decisions, and modify recovery from an acute bleeding episode. Underlying health status is an important determinant of prognosis, but the clinical heterogeneity arising from different combinations of chronic conditions is not adequately represented by conventional risk scores [4,5].

Comorbidity is associated with greater complexity of care, higher health care use, and poorer clinical outcomes among hospitalized patients [6,7]. Rather than appearing at random, chronic conditions tend to cluster into specific patterns or “phenotypes” driven by common risk factors or biological mechanisms. Recent studies have applied unsupervised machine learning approaches to identify comorbidity phenotypes across a range of chronic conditions, including chronic obstructive pulmonary disease and inflammatory bowel disease [8,9]. These data-driven approaches can group patients into clinically meaningful subtypes that traditional clinical classifications often overlook. Such phenotypes have been associated with differences in health care use, disease progression, and mortality across several patient populations.

Despite these advances, patterns of coexisting chronic conditions among patients with NVUGIB and their associations with clinical outcomes remain poorly understood. We therefore conducted a retrospective cohort study of patients hospitalized with NVUGIB between 2015 and 2025. Using unsupervised machine learning, we aimed to identify clinically interpretable comorbidity profiles, evaluate the reproducibility of their composition, and examine their associations with clinical outcomes and resource use as a formative basis for future multicenter validation.

## Methods

### Data Source and Study Population

This retrospective, hospital-based cohort study used hospital discharge records (HDRs) from the China-Japan Friendship Hospital. We screened all hospitalizations of adults aged 18 years or older who were admitted between January 1, 2015, and December 31, 2025. Each HDR contained an anonymized patient identifier, one primary discharge diagnosis, up to 40 additional discharge diagnoses, corresponding present-on-admission indicators, and information on surgical and nonsurgical procedures. For patients with more than one eligible hospitalization, the first eligible hospitalization observed during the study period was designated as the index hospitalization and retained for analysis. All diagnoses were coded according to the *International Classification of Diseases, 10th Revision* (*ICD-10*), including institution-specific code extensions.

Hospitalizations were considered eligible for the algorithm-defined NVUGIB cohort if at least 1 primary or additional discharge diagnosis met one of the following criteria: (1) upper gastrointestinal bleeding coded as K92.207 or K92.208 according to the Chinese national clinical version of the *ICD-10*; (2) a diagnosis label explicitly indicating “upper gastrointestinal bleeding” or “acute upper gastrointestinal bleeding;” or (3) a lesion-specific nonvariceal source of upper gastrointestinal bleeding, including gastroduodenal ulcer bleeding, hemorrhagic gastric mucosal disease, Dieulafoy lesions, Mallory-Weiss syndrome, or upper gastrointestinal malignancy-related bleeding. For hospitalizations coded as K92.207 or K92.208 without a lesion-specific diagnosis, the complete medical record was reviewed for targeted case adjudication. Apart from this targeted review, endoscopy reports were not systematically reviewed for all patients; therefore, the cohort was described as having algorithm-defined NVUGIB.

For gastroduodenal ulcer codes K25 to K28, the corresponding diagnosis label was additionally required to indicate bleeding explicitly. Irrespective of diagnostic position, hospitalizations were excluded if any diagnosis or procedure indicated a potential variceal source, including an *ICD-10* code beginning with I85.0 or I86.4; a liver disease–variceal combination code containing I98.3; a diagnosis label indicating esophageal, gastric, or gastroesophageal varices; or a procedure involving variceal ligation, cyanoacrylate injection, or sclerotherapy. Hospitalizations were also excluded if any discharge diagnosis suggested a potential lower gastrointestinal bleeding source that was inconsistent with classification as NVUGIB. Such hospitalizations were identified either by a structured *ICD-10* code (K92.209, K92.200, or K92.204) or by diagnosis-label text indicating lower gastrointestinal bleeding, hematochezia, or intestinal bleeding. Cirrhosis or portal hypertension alone was not an exclusion criterion in the absence of documented varices or variceal treatment. Because case ascertainment was based primarily on HDRs and medical records were reviewed only for targeted adjudication rather than systematically for all patients, the study population was described as having algorithm-defined NVUGIB.

### Ethical Considerations

This study was conducted in accordance with the Declaration of Helsinki and was approved by the ethics review committees of the China-Japan Friendship Hospital (2024-KY-405). The requirement for informed consent was waived by the ethics committee due to the retrospective and anonymized nature of this research.

### Measurements

Variables of interest included demographic characteristics, such as age, sex, occupation, marital status, type of medical insurance, and payment method. Hospitalization-related variables included length of hospital stay and hospital expenditure.

The primary outcomes were in-hospital death and severe in-hospital outcomes. A severe in-hospital outcome was defined as the occurrence of at least one of the following: invasive mechanical ventilation, continuous renal replacement therapy, or vasoactive drug support. Other outcomes included intensive care unit (ICU) admission, length of hospital stay, and total hospital costs. Invasive mechanical ventilation and continuous renal replacement therapy were identified from relevant procedure codes in HDRs, physician orders, or clinical progress notes; vasoactive drug support was identified from physician orders; and ICU admission was determined from structured ICU admission records. Length of hospital stay was measured in days, and total hospital costs were reported in Chinese yuan (CN ¥ 1=US $0.15 as of September 22, 2026).

### Definitions of Comorbidities

A total of 55 major physical and mental diseases spanning 17 organ and body systems were identified. The initial candidate list was developed with reference to conditions contributing substantially to disability-adjusted life years and mortality or having a high prevalence in China, as reported in the Global Burden of Disease Study 2023. The list was subsequently supplemented and refined based on previously published comorbidity frameworks and multidisciplinary expert consensus [10-13]. Discharge diagnoses within the HDRs were screened using *ICD-10* codes from chapters I to XIV and XVI to XVIII [14]. Following World Health Organization guidelines, specific diseases were assigned 3- or 4-character *ICD-10* codes. The complete list of candidate comorbidities, disease categories, and corresponding ICD-10 codes is provided in Table S1 in Multimedia Appendix 1. Blank additional diagnosis fields were treated as unused diagnosis slots and were ignored. Each condition was coded as 1 when a corresponding *ICD-10* code was recorded in any available diagnosis field and as 0 otherwise. A value of 0 indicated the absence of a recorded code rather than confirmed absence of the condition. No imputation was performed.

### Unsupervised Machine Learning

Before clustering, the patient-level prevalence of each of the 55 candidate conditions was calculated in the overall study cohort, with all eligible patients included in the denominator. To reduce the influence of sparsely represented variables, only conditions with a prevalence of at least 1% were retained. Thirty-three conditions met this prespecified threshold and were included as binary variables in the clustering analysis. The remaining 22 conditions were excluded only as clustering variables; patients with these conditions remained in the study cohort.

Using a fixed random seed of 20,260,320, the cohort was randomly divided into a training set (2554/3193, 80%) and a held-out validation set (639/3193, 20%) before clustering. Patients with none of the 33 selected comorbidities were assigned to cluster 0 and excluded from self-organizing map (SOM) training. Among training set patients with at least 1 selected comorbidity, the binary comorbidity indicators were entered without additional transformation or weighting into an SOM implemented using the *kohonen* package in R (R Foundation for Statistical Computing). The SOM comprised a 20 × 17 hexagonal grid (340 nodes) and was trained for 1000 iterations, with the learning rate decreasing from 0.05 to 0.01 (Figure S1 in Multimedia Appendix 1). The same fixed random seed was used for SOM training.

Euclidean distances between the SOM node codebook vectors were analyzed using Ward D2 hierarchical clustering. Candidate solutions ranging from 2 to 10 clusters were evaluated using 20 internal validity indexes implemented in *NbClust*. The indexes produced heterogeneous recommendations, with 2, 3, 6, 7, and 10 clusters receiving the joint highest number of recommendations (3 indexes each). Hierarchical cluster assignments were generated for every candidate value of *K*, and the resulting solutions were compared with respect to cluster size, comorbidity composition, clinical interpretability, and parsimony. The 6-cluster solution was retained because it provided more clinically coherent profiles while maintaining a relatively small number of clusters. It was treated as a supported and clinically interpretable solution rather than a uniquely optimal solution. The internal validity assessment and Ward D2 hierarchical clustering dendrogram are presented in Figure S2 in Multimedia Appendix 1.

### Cluster Validation

The reproducibility of the comorbidity profiles was evaluated in the held-out validation set without retraining the SOM, re-estimating the Ward D2 hierarchy, or using clinical outcomes. Validation set patients with none of the selected comorbidities were assigned to cluster 0. All other patients were mapped to their best-matching nodes in the fixed training SOM, and their profile assignments were determined by the corresponding Ward D2 node memberships.

The prevalence of each of the 33 selected comorbidities was calculated within each profile in the training and validation sets. For each profile, the corresponding prevalence vectors were compared using Spearman correlation coefficients and mean absolute differences (MADs), expressed in percentage points. Spearman correlations assessed concordance in the relative ordering of conditions, whereas MADs quantified absolute differences in prevalence. Values closer to 1 for the Spearman correlation and closer to 0 for the MAD indicated greater reproducibility.

Uncertainty was estimated using 1000 patient-level bootstrap samples drawn with replacement from the validation set while keeping the training profiles and fitted SOM–Ward D2 solution fixed. Empirical 95% CIs were calculated using the 2.5th and 97.5th percentiles of the bootstrap distributions. An overall profile reproducibility statistic was calculated as the validation profile size–weighted mean of the profile-specific Spearman correlations and compared with a null distribution generated from 1000 permutations of the validation set profile labels. A fixed random seed of 20,260,730 was used for the bootstrap and permutation analyses. As a complementary assessment of patient-level cluster separation, silhouette coefficients were calculated in the validation set using asymmetric binary (Jaccard) distances. Cluster 0 was excluded from this assessment because it was structurally defined by the absence of all selected comorbidities.

### Statistical Analysis

Continuous variables are presented as medians and IQRs, and categorical variables are presented as frequencies and percentages. Comparisons between patients with and without selected comorbidities were performed using the Wilcoxon rank-sum test for continuous variables, whereas comparisons across comorbidity profiles were performed using the Kruskal-Wallis test. Categorical variables were compared using the Pearson chi-square test.

Multivariable logistic regression was used to examine associations between comorbidity profiles and the binary outcomes of in-hospital death, severe in-hospital outcomes, and ICU admission during the index hospitalization. The models estimated covariate-adjusted associations on the odds scale, reported as adjusted odds ratios (aORs) with 95% CIs. These estimates represent odds ratios rather than risk ratios and should not be interpreted as relative changes in outcome probability, particularly for common outcomes. Length of hospital stay was analyzed using negative binomial regression and is reported as an adjusted incidence rate ratio with a 95% CI. Hospital costs were analyzed using a gamma regression model with a log link and are reported as adjusted cost ratios with 95% CIs. Patients without any of the selected comorbidities served as the reference group.

All models were adjusted for age, sex, systolic blood pressure, hemoglobin concentration (g/L), and urea concentration (mmol/L); the hospital cost model was additionally adjusted for year of admission. Continuous adjustment variables were standardized before model fitting. Missing data were handled using complete-case analysis separately for each model, and the gamma regression analysis was restricted to patients with positive hospital costs. In the dataset used for the current outcome analyses, each adjusted model included 2554 patients.

All tests were 2 sided, and *P*<.05 was considered statistically significant. Analyses were performed using R (version 4.4.3). Key packages included *kohonen* for SOM analysis, *NbClust* for cluster number assessment, *MASS* for negative binomial regression, *broom* for model estimates, and *cluster* for cluster validation.

## Results

### Patient Characteristics

The source data comprised 4611 hospitalizations among 3523 patients. Of the 4611 hospitalizations, 30 (0.7%) involving patients younger than 18 years and 717 (15.5%) involving suspected variceal bleeding were excluded. The remaining 3864 hospitalizations (83.8%) involved 3193 of the initial 3523 patients (90.6%). After repeat hospitalizations were removed, the final cohort comprised 3193 unique patients who were randomly allocated to the training set (n=2554, 80%) and held-out validation set (n=639, 20%; Figure 1).

**Figure 1.**
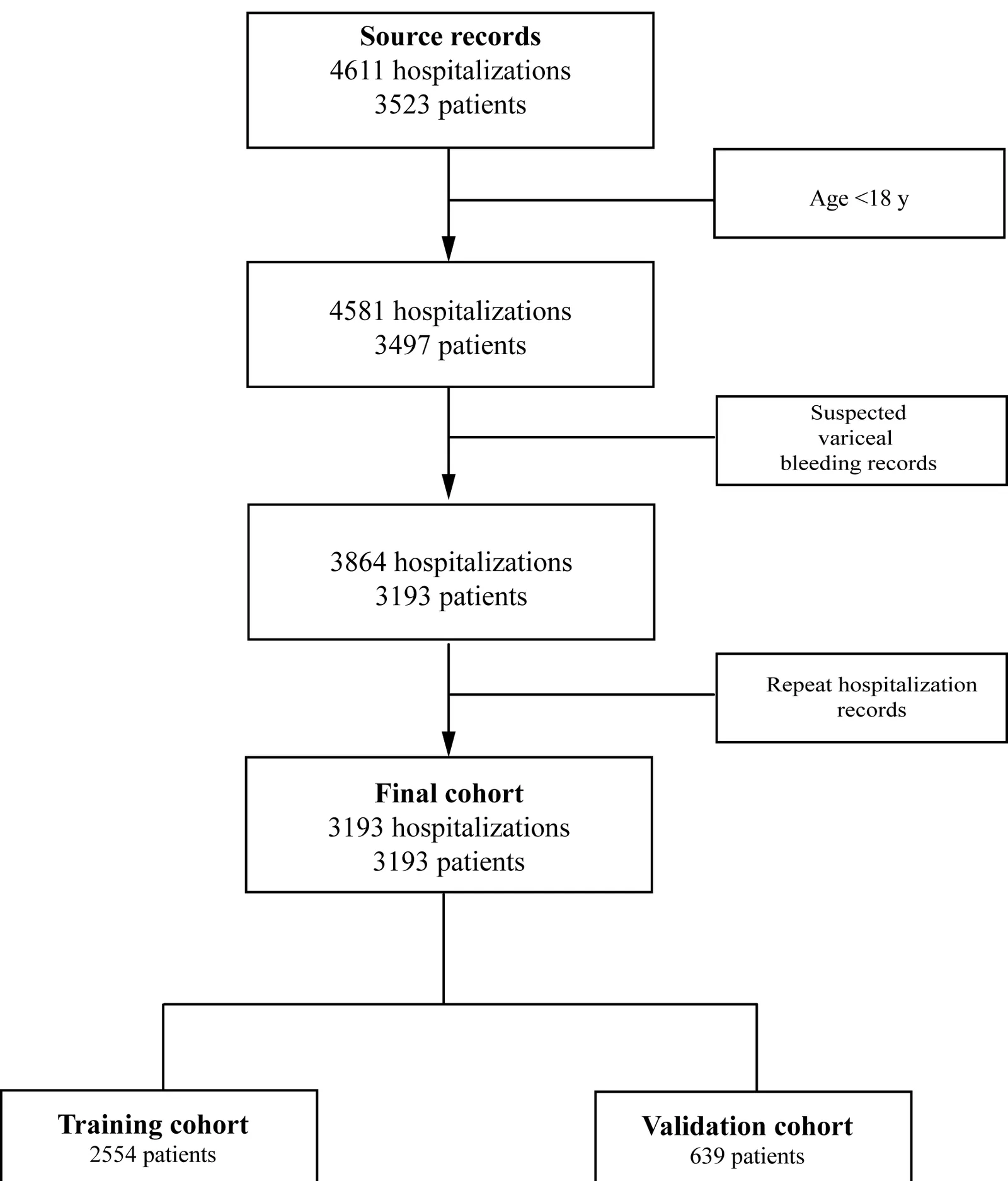
Flowchart of cohort formation and allocation**.** The source data comprised 4611 hospitalizations among 3523 patients. Records involving patients aged younger than 18 years, suspected variceal bleeding, or repeat hospitalizations were excluded. The final cohort comprised 3193 unique patients and was randomly divided into a training cohort (n=2554) and a held-out validation cohort (n=639).

The principal bleeding etiologies were peptic ulcer bleeding (2009/3193, 62.9%), gastrointestinal tumors (515/3193, 16.1%), and other causes (669/3193, 21%). At least 1 selected comorbidity was recorded in 89.7% (2864/3193) of the patients, whereas 10.3% (329/3193) had none. Overall, 15.3% (487/3193) of the patients had 1 selected comorbidity, 18.4% (587/3193) had 2, and 56.1% (1790/3193) had 3 or more; 74.4% (2377/3193) of the patients had at least 2 (Figure S3 in Multimedia Appendix 1).

Compared with patients with no selected comorbidities, those with at least 1 were older (median 68.0, IQR 58.0-77.0 years vs median 53.0, IQR 36.0-67.0 years; *P*<.001) and more frequently female (917/2864, 32% vs 81/329, 24.6%; *P*=.007). The median GBS was 8.0 in both groups, although its distribution differed (IQR 6.0-11.0 vs IQR 6.0-9.0 in the groups with and without comorbidities, respectively; *P*<.001). Patients with at least 1 selected comorbidity had higher proportions of in-hospital death (283/2864, 9.9% vs 7/329, 2.1%), severe in-hospital outcomes (1123/2864, 39.2% vs 39/329, 11.9%), and ICU admission (304/2864, 10.6% vs 8/329, 2.4%; *P*<.001 in all cases). They also had a longer median hospital stay (11.0, IQR 7.0-18.0 vs 7.0, IQR 5.0-11.0 days) and higher median total hospital costs (CN¥ 35,098.90, IQR CN¥ 14,944.50-95,381 vs CN¥ 12,710.20, IQR CN¥ 7397.50-37,261.60; *P*<.001 in both cases; Table 1).

**Table 1.** Patient characteristics and in-hospital outcomes according to selected comorbidity status (N=3193)^a^.

Characteristics	With comorbidity (n=2864)	Without comorbidity (n=329)	*P* value
Age (y), median (IQR)	68.0 (58.0-77.0)	53.0 (36.0-67.0)	<.001
Sex, n (%)			.007
Male	1947 (68)	248 (75.4)	
Female	917 (32)	81 (24.6)	
GBS^b^, median (IQR)	8.0 (6.0-11.0)	8.0 (6.0-9.0)	<.001
Deaths, n (%)	283 (9.9)	7 (2.1)	<.001
Length of stay (d), median (IQR)	11.0 (7.0-18.0)	7.0 (5.0-11.0)	<.001
Severe in-hospital outcomes, n (%)	1123 (39.2)	39 (11.9)	<.001
ICU^c^ admission, n (%)	304 (10.6)	8 (2.4)	<.001
Hospitalization cost (CN ¥; CN ¥ 1=US $0.15 as of September 22, 2026), median (IQR)	35,098.90 (14,944.50-95,381)	12,710.20 (7397.50-37,261.60)	<.001

a Severe in-hospital outcome was defined as the receipt of invasive mechanical ventilation, continuous renal replacement therapy, or vasoactive drug support. *P* values were calculated using the Wilcoxon rank-sum test for continuous variables and the Pearson chi-square test for categorical variables.

b GBS: Glasgow-Blatchford Score.

### Prevalence of Comorbidities

Comorbidity prevalence varied by sex (Figure 2). Diabetes mellitus was the most prevalent condition among both female (316/998, 31.7%) and male (616/2195, 28.1%) patients. Other prevalent conditions among female patients included hypertension, heart failure, hyperlipidemia, and solid tumors, whereas ischemic heart disease, hypertension, stroke, and solid tumors were common among male patients. Female patients had relatively higher prevalence of heart failure, atrial fibrillation, osteoporosis, and connective tissue disease, whereas male patients had higher prevalence of ischemic heart disease, chronic liver disease, hyperuricemia, and metastatic malignancy.

**Figure 2.**
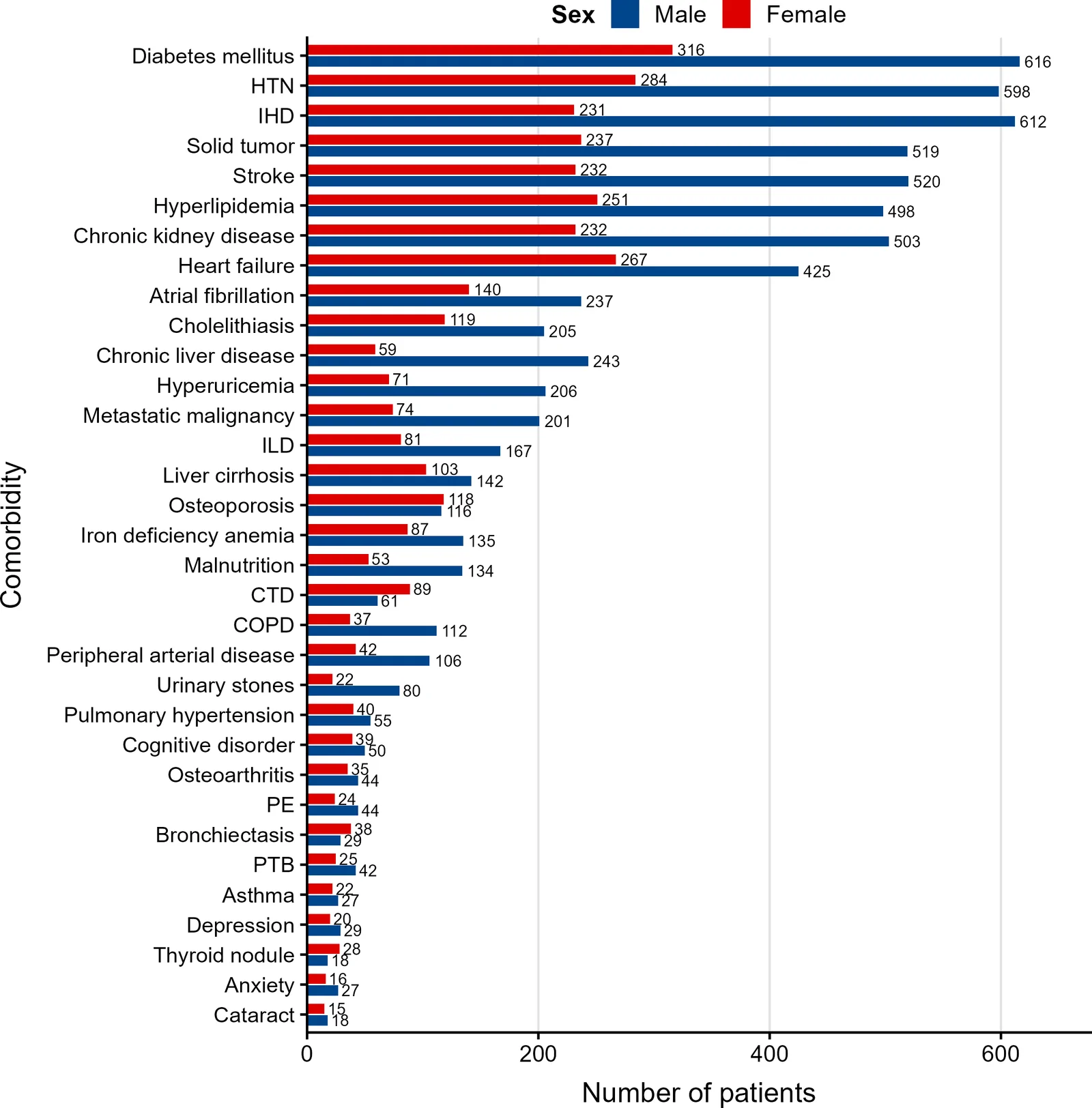
Sex-specific prevalence of common comorbidities among patients with nonvariceal upper gastrointestinal bleeding. COPD: chronic obstructive pulmonary disease; CTD: connective tissue disease; HTN: hypertension; IHD: ischemic heart disease; ILD: interstitial lung disease; PE: pulmonary embolism; PTB: pulmonary tuberculosis.

### Identification of Comorbidity Profiles

Of the 55 prespecified chronic conditions, 33 had a prevalence of at least 1% and were retained for profile identification. The prevalence of each condition across the 6 data-derived profiles is shown in Figure 3. The heat map shows that the profiles were characterized by different combinations of conditions rather than by comorbidity burden alone. The internal validity indexes did not identify a single preferred number of clusters. *K*=2, *K*=3, *K*=6, *K*=7, and *K*=10 each received 3 recommendations, representing the joint highest count. After all candidate solutions from *K*=2 to *K*=10 were generated and reviewed, *K*=6 was selected because it retained a relatively parsimonious structure and yielded clinically interpretable comorbidity profiles (Multimedia Appendix 1).

**Figure 3.**
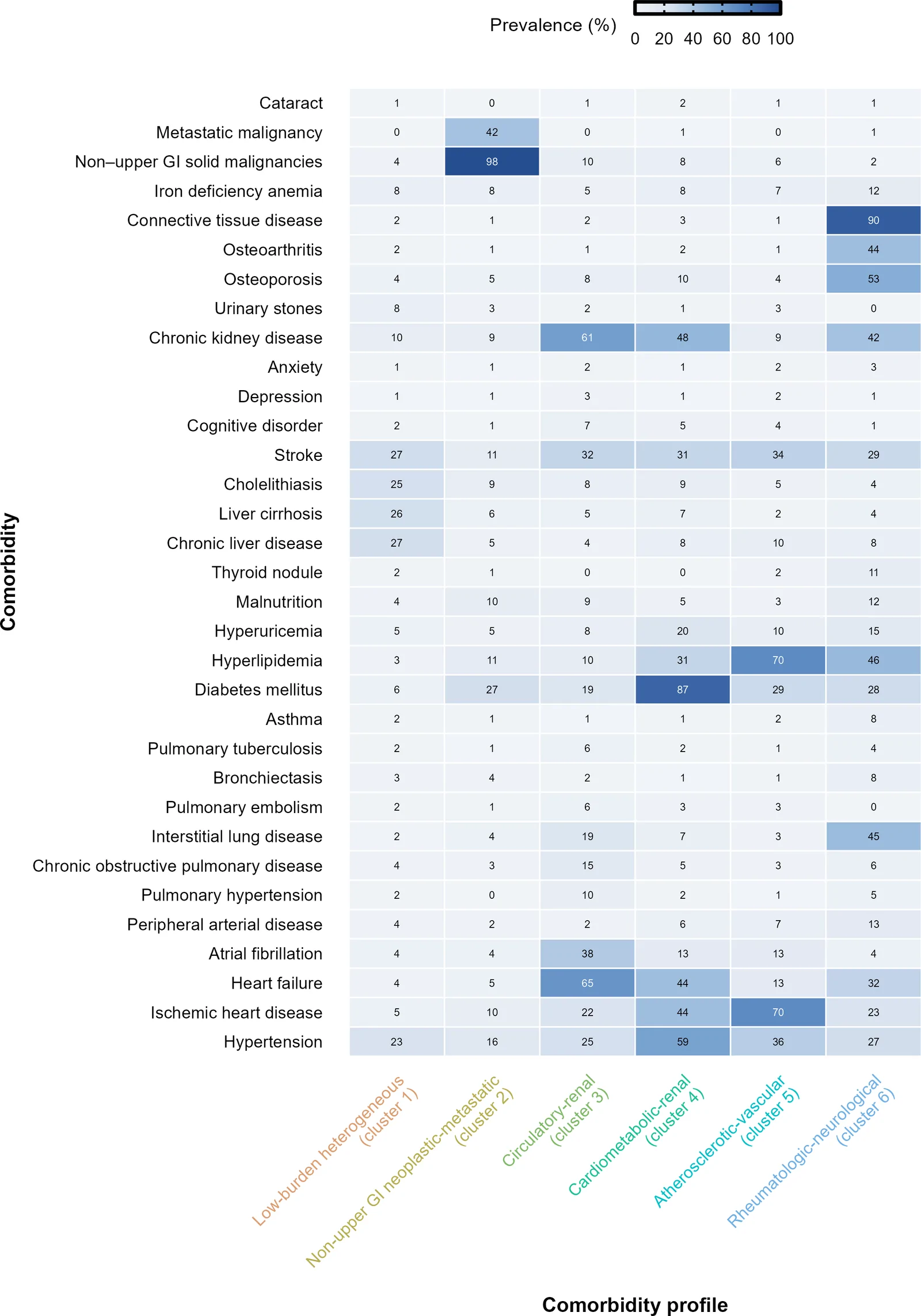
Prevalence of the 33 selected comorbidities across the 6 comorbidity profiles. Rows represent the selected comorbidities, and columns represent the 6 profiles identified in the training cohort. Values within cells are the percentages of patients in each profile with the corresponding comorbidity; darker shading indicates a higher prevalence. The analysis included 2291 training set patients with at least 1 selected comorbidity. Profile sizes were 460 for the low-burden heterogeneous profile, 490 for the non–upper gastrointestinal neoplastic-metastatic profile, 367 for the circulatory-renal profile, 410 for the cardiometabolic-renal profile, 471 for the atherosclerotic-vascular profile, and 93 for the rheumatologic-neurological profile. Cluster 0 was excluded because it was defined by the absence of all selected comorbidities. GI: gastrointestinal.

The low-burden heterogeneous profile (cluster 1) had the lowest overall comorbidity burden, with dispersed occurrences of stroke, chronic liver disease, and liver cirrhosis. The non–upper gastrointestinal neoplastic-metastatic profile (cluster 2) was characterized by non–upper gastrointestinal solid tumors (479/490, 97.8%) and metastatic malignancy (205/490, 41.8%). The circulatory-renal profile (cluster 3) was dominated by heart failure (239/367, 65.1%) and chronic kidney disease (223/367, 60.8%), frequently accompanied by atrial fibrillation and stroke. The cardiometabolic-renal profile (cluster 4) included high prevalences of diabetes mellitus (358/410, 87.3%), hypertension (240/410, 58.5%), and chronic kidney disease (196/410, 47.8%). The atherosclerotic-vascular profile (cluster 5) was characterized by ischemic heart disease and hyperlipidemia (both 329/471, 69.9%), whereas the rheumatologic-neurological profile (cluster 6) was characterized by connective tissue disease (84/93, 90.4%), interstitial lung disease (42/93, 45.2%), and osteoporosis (49/93, 53%).

### Patient Characteristics Across Comorbidity Profiles

Patient characteristics and resource use differed across profiles (Table 2). The circulatory-renal profile included the oldest patients (median age 74.0, IQR 63.0-81.0 years). The atherosclerotic-vascular profile had the highest proportion of male patients (354/471, 75.2%) and the shortest median hospital stay among the comorbidity profiles (8.0, IQR 6.0-14.0 days). The rheumatologic-neurological profile was the only female-majority profile (51/93, 54.8%) and had the longest median hospital stay (14.0, IQR 8.0-21.0 days).

**Table 2.** Baseline characteristics across comorbidity profiles in the training cohort.

Variables	Cluster 0 (n=263)	Cluster 1 (n=460)	Cluster 2 (n=490)	Cluster 3 (n=367)	Cluster 4 (n=410)	Cluster 5 (n=471)	Cluster 6 (n=93)	Total (n=2554)
Age (y), median (IQR)	52.0 (35.0-66.0)	64.0 (51.0-75.0)	66.0 (58.0-73.0)	74.0 (63.0-81.0)	70.0 (61.0-80.0)	68.0 (60.0-77.0)	67.0 (55.0-73.0)	67.0 (56.0-76.0)
Sex (male), n (%)	196 (74.5)	317 (68.9)	337 (68.8)	242 (65.9)	268 (65.4)	354 (75.2)	42 (45.2)	1756 (68.8)
GBS^a^, median (IQR)	7.0 (6.0-9.0)	8.0 (6.0-10.0)	6.0 (4.0-9.0)	10.0 (8.0-13.0)	10.0 (7.0-12.0)	7.0 (4.0-10.0)	9.0 (6.0-12.0)	8.0 (6.0-11.0)
In-hospital deaths, n (%)	5 (1.9)	18 (3.9)	40 (8.2)	70 (19.1)	53 (12.9)	21 (4.5)	14 (15.1)	221 (8.7)
Severe in-hospital outcomes, n (%)	28 (10.6)	121 (26.3)	141 (28.8)	239 (65.1)	214 (52.2)	123 (26.1)	52 (55.9)	918 (35.9)
ICU^b^ admission, n (%)	7 (2.7)	23 (5)	10 (2)	89 (24.3)	70 (17.1)	20 (4.2)	19 (20.4)	238 (9.3)
Length of hospital stay (d), median (IQR)	7.0 (5.0-11.0)	9.0 (6.0-14.0)	13.0 (7.0-19.0)	13.0 (7.0-22.0)	12.0 (7.0-21.0)	8.0 (6.0-14.0)	14.0 (8.0-21.0)	10.0 (6.0-17.0)
Total hospital costs (CN¥; CN¥ 1=US $0.15 as of September 22, 2026), median (IQR)	13,070.60 (7443.6-40,554.60)	20,676.70 (10,025.40-61,810.50)	40,699.50 (20,084.70-89,895.70)	66,554.10 (24,389.80-190,162.60)	47,140.10 (19,035-114,777.70)	21,074.10 (10,420.60-55,579.20)	64,504.60 (20,585-138,670.50)	31,328.50 (12,914.80-89,394.60)

a GBS: Glasgow-Blatchford Score.

b ICU: intensive care unit.

### Reproducibility of Comorbidity Profiles

The training and validation sets included 2291 and 573 patients, respectively, with at least 1 selected comorbidity. The relative sizes of the 6 profiles were broadly similar between the sets. Profile-specific comorbidity prevalence vectors had a weighted mean Spearman correlation of 0.891 (SD 0.04), which exceeded that expected after permutation of the validation set profile labels (*P*=.001). Profile-specific correlations ranged from 0.783 to 0.940, and MADs in prevalence ranged from 1.59 to 6.58 percentage points. The mean silhouette coefficient in the validation set was 0.085 (SD 0.130), with profile-specific means ranging from −0.001 to 0.212.

### Associations Between Comorbidity Profiles and Clinical Outcomes

Adjusted analyses included 2554 patients and used patients with none of the selected comorbidities as the reference group (Figure 4 and Table S2 in Multimedia Appendix 1).

All 6 profiles were associated with higher odds of a severe in-hospital outcome. The strongest associations were observed for the circulatory-renal (aOR 8.57, 95% CI 5.39-14.01), rheumatologic-neurological (aOR 6.68, 95% CI 3.68-12.34), and cardiometabolic-renal profiles (aOR 5.31, 95% CI 3.38-8.59; *P*<.001 in all cases). The low-burden heterogeneous (aOR 2.58, 95% CI 1.66-4.14), non-upper gastrointestinal neoplastic-metastatic (aOR 2.88, 95% CI 1.85-4.60), and atherosclerotic-vascular profiles (aOR 2.31, 95% CI 1.47-3.72) were also associated with higher odds of a severe in-hospital outcome (all *P*<.001).

The circulatory-renal (aOR 10.38, 95% CI 4.87-25.76), rheumatologic-neurological (aOR 9.01, 95% CI 3.71-24.27), and cardiometabolic-renal profiles (aOR 6.79, 95% CI 3.18-16.84; *P*<.001 in all cases) were associated with higher odds of ICU admission. Associations with ICU admission were not statistically significant for the low-burden heterogeneous (aOR 1.90, 95% CI 0.84-4.89; *P*=.15), non-upper gastrointestinal neoplastic-metastatic (aOR 0.77, 95% CI 0.29-2.16; *P*=.60), or atherosclerotic-vascular profiles (aOR 1.57, 95% CI 0.67-4.10; *P*=.32).

Four profiles were associated with higher odds of in-hospital death: circulatory-renal (aOR 4.66, 95% CI 1.97-13.75; *P*=.002), rheumatologic-neurological (aOR 4.43, 95% CI 1.58-14.40; *P*=.007), cardiometabolic-renal (aOR 3.34, 95% CI 1.40-9.88; *P*=.01), and non–upper gastrointestinal neoplastic-metastatic (aOR 2.94, 95% CI 1.24-8.67; *P*=.03). Associations with death were not significant for the low-burden heterogeneous or atherosclerotic-vascular profiles.

All profiles were associated with longer hospital stays and higher total hospital costs. Adjusted incidence rate ratios for length of stay ranged from 1.15 for the atherosclerotic-vascular profile to 1.75 for the rheumatologic-neurological profile. The largest adjusted cost ratios were observed for the circulatory-renal (3.22, 95% CI 2.55-4.06), rheumatologic-neurological (2.96, 95% CI 2.12-4.19), and cardiometabolic-renal (2.41, 95% CI 1.91-3.02; *P*<.001 in all cases) profiles.

**Figure 4.**
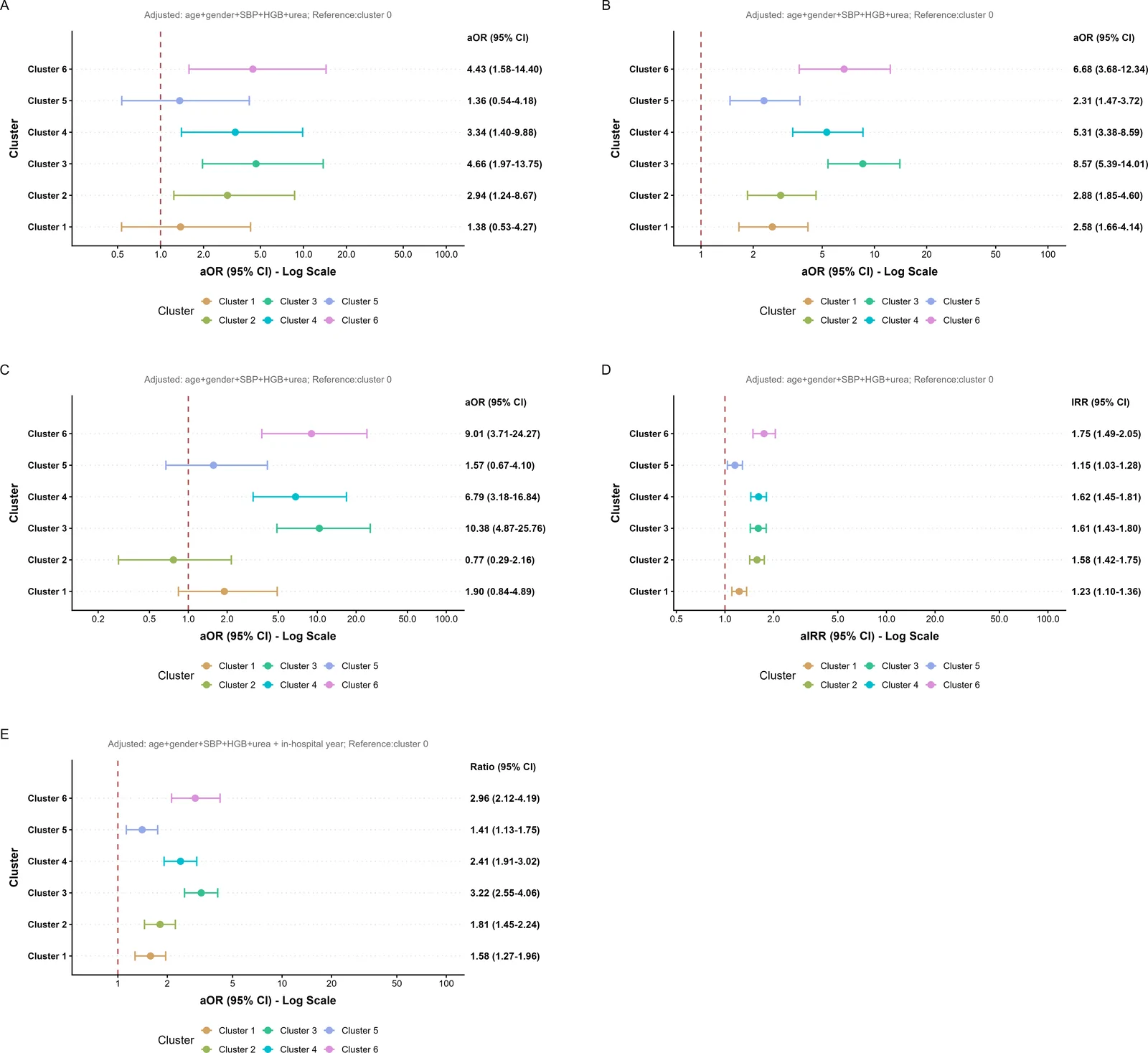
Adjusted associations between comorbidity profiles and clinical outcomes and health care utilization. (A) In-hospital death. (B) Severe in-hospital outcomes. (C) ICU admission. (D) Length of stay. (E) Total hospital costs.

## Discussion

### Principal Findings

In this retrospective study of 3193 patients hospitalized with NVUGIB, 89.7% (n=2864) had at least 1 of the selected comorbidities recorded. Using an SOM followed by Ward D2 hierarchical clustering, we identified 6 clinically interpretable comorbidity profiles: low-burden heterogeneous, non–upper gastrointestinal neoplastic-metastatic, circulatory-renal, cardiometabolic-renal, atherosclerotic-vascular, and rheumatologic-neurological. The profile composition was broadly reproducible in the 20% held-out validation subset, with a weighted mean Spearman correlation of 0.891 between the training and validation subsets. The profiles showed heterogeneous associations with clinical outcomes. The circulatory-renal, cardiometabolic-renal, and rheumatologic-neurological profiles were associated with higher odds of in-hospital death, severe in-hospital outcomes, and ICU admission. The non–upper gastrointestinal neoplastic-metastatic profile was associated with higher odds of in-hospital death and severe in-hospital outcomes, but its association with ICU admission was not statistically significant. The low-burden heterogeneous and atherosclerotic-vascular profiles were associated with higher odds of severe in-hospital outcomes, whereas their associations with in-hospital death and ICU admission were not statistically significant. All 6 profiles were associated with longer hospital stays and higher hospital costs compared with patients without recorded comorbidities.

Previous studies of NVUGIB have primarily focused on established risk scores, individual prognostic factors, rebleeding, and mortality [1-5]. Commonly used scores represent comorbidity using selected conditions or aggregated measures. For example, the GBS includes liver disease and heart failure, the Rockall score uses predefined categories of major comorbidity, and the CHAMPS score incorporates overall comorbidity burden using a threshold based on the Charlson Comorbidity Index [4,15,16]. Although these approaches confirm the prognostic importance of comorbidity, they do not explicitly characterize how individual conditions co-occur. Electronic health record (EHR)–based studies in general patient populations and in patients with chronic obstructive pulmonary disease, inflammatory bowel disease, and COVID-19 have shown that clustering methods can identify clinically meaningful patterns of multimorbidity [8,9,17]. The present study extends this literature to NVUGIB by demonstrating that the clinical implications of comorbidity may depend not only on the presence or number of conditions but also on how those conditions occur together. It also links these combinations to both adverse clinical outcomes and health care use in an acute gastrointestinal bleeding population.

The observed associations are clinically plausible, although the underlying mechanisms cannot be determined from this study. The associations of the circulatory-renal and cardiometabolic-renal profiles with adverse outcomes may partly reflect impaired cardiac and renal reserve, reduced tolerance of acute blood loss, metabolic and microvascular dysfunction, and the effects of antithrombotic therapy [18-29]. The rheumatologic-neurological profile may reflect a combination of systemic inflammation, frailty, and exposure to medications associated with gastrointestinal injury, including nonsteroidal anti-inflammatory drugs, glucocorticoids, antiplatelet agents, and bisphosphonates [30-35]. The associations between the non–upper gastrointestinal neoplastic-metastatic profile and both in-hospital death and severe in-hospital outcomes may reflect tumor burden, treatment-associated mucosal injury, thrombocytopenia, coagulation abnormalities, or reduced physiological reserve [36-40]. These explanations should be regarded as possible clinical pathways informed by previous evidence rather than causal mechanisms established by the present analysis.

### Informatics and Clinical Implications

Routine EHR data contain information on both individual diagnoses and their patterns of co-occurrence. Previous studies have used these data to examine multimorbidity patterns, clinical outcomes, health care use, and costs [17,41]. Comparisons of count-based and cluster-based definitions indicate that the two approaches describe different aspects of multimorbidity. Disease counts measure overall burden, whereas clustering can identify groups with different combinations of conditions and outcome risks. In this study, an SOM followed by Ward D2 hierarchical clustering summarized 33 conditions into 6 clinically interpretable profiles. Their composition was assessed in a held-out subset. The analysis shows how routinely recorded diagnoses can be organized into disease-specific comorbidity profiles and linked to clinical and resource-related outcomes in patients with NVUGIB.

The profiles provide information that is not conveyed by examining each comorbidity separately or by summing the number of conditions. They identify groups of related conditions that may warrant attention to different aspects of clinical care, such as cardiac and renal function, metabolic status, medication exposure, cancer-related factors, and hematologic abnormalities. This approach could allow clinicians to focus on the most relevant comorbidity domains instead of assigning equal importance to every recorded condition. For example, a patient admitted with NVUGIB who reports a history of chronic heart failure and chronic kidney disease may have a comorbidity pattern resembling the circulatory-renal profile, which was associated with higher odds of adverse in-hospital outcomes in this study. Recognition of this pattern could prompt closer assessment of cardiac and renal function and careful monitoring of fluid balance during resuscitation, with attention to restoring perfusion while avoiding fluid overload that could exacerbate heart failure. This example illustrates how comorbidity profiles might complement established bleeding risk scores and clinical judgment. Previous studies have also found that health care use and costs vary across disease combinations and may be greater than the sum of costs associated with individual conditions [42,43]. In the present study, all 6 profiles were associated with longer hospital stays and higher hospital costs. Comorbidity profiles may therefore offer useful information for planning monitoring, multidisciplinary assessment, bed use, and intensive care resources. They should be considered alongside established risk scores and clinical assessment rather than as replacements for these approaches.

### Limitations

This retrospective study was conducted at a single tertiary hospital. Differences in patient characteristics, coding practices, and treatment protocols may limit the applicability of the identified profiles to other settings. The held-out subset was drawn from the same hospital population and, therefore, assessed internal reproducibility only. External validation across countries and in both community and tertiary hospitals is needed before the profiles can be considered generalizable. Comorbidities identified from discharge diagnosis codes may be underrecorded, and the completeness of coding may differ between institutions. The binary indicators used in this study also do not capture disease severity or control. These limitations may affect the observed prevalence and co-occurrence of conditions, influencing the composition of the profiles and their reproducibility in other settings. Although selected diagnostically uncertain cases underwent record review, endoscopy reports were not systematically reviewed for all patients, limiting verification of bleeding sources and endoscopic findings. The observed associations between comorbidity profiles and clinical outcomes should not be interpreted as causal.

### Conclusions

Using routinely collected EHR data, we identified 6 clinically interpretable comorbidity profiles among patients hospitalized with NVUGIB. The profiles were broadly reproducible within the study population and showed different associations with mortality, severe in-hospital outcomes, ICU admission, length of stay, and hospital costs. Comorbidity patterns may provide information on clinical and resource-related differences beyond that obtained from individual diagnoses or simple disease counts. Profile-based information could be evaluated as a potential complement to established risk assessment and inform multidisciplinary management and hospital resource planning.

## Supplementary material

10.2196/108793 Multimedia Appendix 1 Supplementary methods, tables, and figures describing comorbidity definitions, self-organizing map configuration, cluster selection and validation, and outcome analyses.

## Abbreviations

aOR: adjusted odds ratio
EHR: electronic health record
GBS: Glasgow-Blatchford Score
HDR: hospital discharge record
ICD-10: International Classification of Diseases, 10th Revision
ICU: intensive care unit
MAD: mean absolute difference
NVUGIB: nonvariceal upper gastrointestinal bleeding
SOM: self-organizing map
