# Supplementary material for: Data-Driven Comorbidity Phenotypes and In-Hospital Outcomes Among Patients With Nonvariceal Upper Gastrointestinal Bleeding: Formative Retrospective Cohort Study
Source: JMIR Form Res. 2026 Oct 19;10:e108793. doi: 10.2196/108793 (PMC13684116; doi:10.2196/108793)
Supplement: Supplementary file 1 [file formative-v10-e108793-s001.docx]

**Supplementary Material**

**Supplementary Methods**

Configuration of the self-organizing maps, the process for determining optimal cluster assignment, and additional stability testing of the clusters

**Table S1** Included comorbidities with ICD-10 codes and disease categories

**Table S2.** Unadjusted and adjusted associations between comorbidity profiles and in-hospital outcomes and health care utilization in the training cohort (n=2554).

**Table S3.** Cluster Numbers Recommended by the Internal Validity Indices

**Figure S1** The progress of the mean distance to the closest unit across 500, 750, 1000, 1200, and 1500 iterations.

**Figure S2** Internal validity assessment across candidate cluster solutions and dendrogram’s structure of the hierarchical clustering.

**Figure S3** Distribution of patients with different number of comorbidities in the total cohort

**Supplementary Methods**

**1. Configuration of the self-organizing maps, the process for determining optimal cluster assignment, and additional stability testing of the clusters**

Self-organizing maps (SOMs) were implemented by R version 4.4.3 using the publicly available R library: kohonen1 (v.3.0.11). Clusters were superimposed onto the SOMs using the Ward.D2 (Ward Cluster) algorithm. Default hyperparameters were applied, with modifications to grid size (grid), iteration length (rlen), and the number of clusters (k_cluster). The grid was calculated using the heuristic formula:5 × n^0.54321^, where n represents the number of records in the dataset. The rlen and k_cluster parameters were optimized for our cohort, as detailed in sections 2.1.1 and 2.1.2.

**1.1 SOMs for our cohort**

Optimized parameters used in the SOM: grid=(20,17), rlen=1000, k_cluster=6.

**1.1.1 Iteration length (rlen) optimization**

During SOM training, the distance between each node's weights and the samples it represents decreases progressively with each iteration over the full dataset. This distance should stabilize at a minimum plateau, beyond which further iterations yield negligible improvements. Monitoring this progress over time through a plot can indicate whether convergence has been achieved. If the curve continues to decrease, additional iterations are necessary.

See Figure S1 for monitoring of the progress.

To determine the training length of the SOM, we compared convergence plots across several iteration settings. The mean distance to the closest unit decreased substantially during the initial training phase and showed no meaningful further reduction after 1,000 iterations. The cluster patterns were also visually stable when higher values were tested. Based on these findings, rlen was set to 1,000 for the final SOM model.

**Figure S1** The progress of the mean distance to the closest unit across 500, 750, 1000, 1200, and 1500 iterations.

**1.1.2 Assessment and Selection of the Number of Clusters**

To assess the number of clusters, we applied NbClust to the self-organizing map codebook vectors using Euclidean distance and Ward.D2 linkage. Candidate solutions ranging from K=2 to K=10 were evaluated using 20 internal validity indices. K=2, K=3, K=6, K=7, and K=10 each received 3 recommendations, the joint highest count. Hierarchical cluster assignments were generated for every candidate value of K. The solutions were compared with respect to cluster size, the distribution of the 33 comorbidities, clinical coherence, and parsimony. The 6-cluster solution was retained because it produced more clinically interpretable comorbidity profiles while maintaining a relatively small number of clusters. It was therefore considered a supported and clinically interpretable solution rather than a uniquely optimal solution. The distribution of recommendations across the candidate cluster solutions is shown in Figure S2A, and the corresponding recommendation counts are reported in Table S3. The Ward.D2 hierarchical clustering dendrogram is shown in Figure S2B.

**Figure S2. Assessment of candidate cluster solutions and hierarchical clustering of the SOM nodes.** (A) Number of internal validity indices recommending each candidate solution from K=2 to K=10. Candidate solutions were evaluated using 20 indices implemented in NbClust. K=2, K=3, K=6, K=7, and K=10 each received 3 recommendations, representing the joint highest count. The recommendations did not identify a uniquely optimal solution. (B) Ward.D2 hierarchical clustering dendrogram of the SOM node codebook vectors. The 6-cluster solution was retained after considering the index recommendations, cluster sizes, comorbidity composition, clinical interpretability, and parsimony.

**Figure S3. Distribution of patients with different number of comorbidities in the total cohort**

**Table S1. Candidate Comorbidities, Disease Categories, and ICD-10 Codes**

| **Disease Category** | **Specific Comorbidity** | **ICD-10 Codes** |
| --- | --- | --- |
| **Respiratory** | Chronic obstructive pulmonary disease | J41-J44 |
| **Respiratory** | Asthma | J45-J46 |
| **Respiratory** | Pulmonary hypertension | I27.0, I27.2 |
| **Respiratory** | Interstitial lung diseases | J60-J65; J67; J70.1-J70.4; J84.0-J84.9; D86.0-D86.2 |
| **Respiratory** | Chronic respiratory failure | J96.1 |
| **Respiratory** | Sleep apnoea | G47.3 |
| **Respiratory** | Pulmonary/respiratory tuberculosis | A15-A16 |
| **Respiratory** | Bronchiectasis | J47 |
| **Cerebral & Cardiovascular** | Heart failure | I50 |
| **Cerebral & Cardiovascular** | Peripheral arterial disease | I70.2; I73.0-I73.1; I73.9 |
| **Cerebral & Cardiovascular** | Pulmonary embolism | I26; I80-I82 |
| **Cerebral & Cardiovascular** | Chronic rheumatic heart disease | I05-I09 |
| **Cerebral & Cardiovascular** | Hypertensive heart disease | I11; I13 |
| **Cerebral & Cardiovascular** | Myocarditis and cardiomyopathy | I40-I43 |
| **Cerebral & Cardiovascular** | Ischaemic heart disease | I20-I25 |
| **Cerebral & Cardiovascular** | Atrial fibrillation and flutter | I48 |
| **Cerebral & Cardiovascular** | Hypertension | I10-I15 |
| **Cerebral & Cardiovascular** | Stroke and sequelae | I60-I64; I69.0-I69.4; I69.8 |
| **Digestive** | Gastro-oesophageal reflux disease | K21 |
| **Digestive** | Peptic ulcer disease | K25-K28 |
| **Digestive** | Fatty liver, not elsewhere classified | K76.0 |
| **Digestive** | Cholelithiasis | K80 |
| **Digestive** | Cirrhosis and fibrosis of liver | K70.3; K71.7; K74.0-K74.6 |
| **Digestive** | Chronic viral hepatitis | B18 |
| **Endocrine** | Diabetes mellitus | E10-E14 |
| **Endocrine** | Disorders of lipoprotein metabolism and other lipidaemias | E78 |
| **Endocrine** | Thyroid diseases | E00-E07 |
| **Endocrine** | Hyperuricaemia without inflammatory arthritis or tophaceous disease | E79.0 |
| **Endocrine** | Obesity | E66 |
| **Endocrine** | Protein-energy malnutrition | E40-E46 |
| **Immunological** | Systemic connective tissue disorders | M30-M36 |
| **Cancers** | Non-upper gastrointestinal solid malignancies | C00-C80; C97; excluding C15, C16 and C81–C96 |
| **Cancers** | Metastatic malignancy | C77-C79 |
| **Genitourinary** | Polycystic ovarian syndrome | E28.2 |
| **Genitourinary** | Chronic kidney disease | N18 |
| **Genitourinary** | Urinary tract calculi | N20-N23 |
| **Genitourinary** | Hyperplasia of prostate | N40 |
| **Haematological** | Anaemias | D50-D64 |
| **Haematological** | Haematological neoplasms | C81-C96; D45-D47 |
| **Musculoskeletal** | Osteoporosis | M80-M81 |
| **Musculoskeletal** | Osteoarthritis | M15-M19 |
| **Neurological** | Dementia and related neurodegenerative disorders | F00-F03; G30-G31 |
| **Neurological** | Parkinson disease and secondary parkinsonism | G20-G21 |
| **Psychiatric** | Depressive disorders | F32-F33; F34.1 |
| **Psychiatric** | Anxiety disorders | F40-F41 |
| **Psychiatric** | Schizophrenia | F20 |
| **Ear, nose, and throat** | Hearing loss | H90-H91 |
| **Ear, nose, and throat** | Rhinitis | J30; J31.0 |
| **Eye** | Cataract | H25-H26 |
| **Eye** | Visual disturbances, blindness and low vision | H53-H54 |
| **Eye** | Glaucoma | H40-H42 |
| **Oral** | Gingivitis and periodontal diseases | K05 |
| **Pain** | Dorsalgia/back pain | M54.0-M54.9 |
| **Pain** | Migraine and other headache syndromes | G43-G44 |
| **skin** | Dermatitis and eczema | L20-L30 |

**Table S2. Unadjusted and adjusted associations between comorbidity profiles and in-hospital outcomes and health care utilization in the training cohort (n=2554).**

| Outcome | Cluster | Unadjusted OR (95% CI) | Unadjusted p | Adjusted OR (95% CI) | Adjusted p | Unadjusted IRR (95% CI) | Adjusted IRR (95% CI) | Unadjusted Ratio (95% CI) | Adjusted Ratio (95% CI) |
| --- | --- | --- | --- | --- | --- | --- | --- | --- | --- |
| Death | Cluster 1 | 2.10 (0.83-6.42) | 0.147 | 1.38 (0.53-4.27) | 0.535 |  |  |  |  |
|  | Cluster 2 | 4.59 (1.96-13.42) | 0.002 | 2.94 (1.24-8.67) | 0.027 |  |  |  |  |
|  | Cluster 3 | 12.16 (5.33-35.09) | <0.001 | 4.66 (1.97-13.75) | 0.002 |  |  |  |  |
|  | Cluster 4 | 7.66 (3.32-22.23) | <0.001 | 3.34 (1.40-9.88) | 0.014 |  |  |  |  |
|  | Cluster 5 | 2.41 (0.97-7.28) | 0.081 | 1.36 (0.54-4.18) | 0.548 |  |  |  |  |
|  | Cluster 6 | 9.14 (3.38-29.02) | <0.001 | 4.43 (1.58-14.40) | 0.007 |  |  |  |  |
| Severe in-hospital outcome | Cluster 1 | 3.00 (1.95-4.75) | <0.001 | 2.58 (1.66-4.14) | <0.001 |  |  |  |  |
|  | Cluster 2 | 3.39 (2.22-5.35) | <0.001 | 2.88 (1.85-4.60) | <0.001 |  |  |  |  |
|  | Cluster 3 | 15.67 (10.17-24.93) | <0.001 | 8.57 (5.39-14.01) | <0.001 |  |  |  |  |
|  | Cluster 4 | 9.16 (6.01-14.44) | <0.001 | 5.31 (3.38-8.59) | <0.001 |  |  |  |  |
|  | Cluster 5 | 2.97 (1.93-4.70) | <0.001 | 2.31 (1.47-3.72) | <0.001 |  |  |  |  |
|  | Cluster 6 | 10.64 (6.10-19.00) | <0.001 | 6.68 (3.68-12.34) | <0.001 |  |  |  |  |
| ICU Admission | Cluster 1 | 1.92 (0.86-4.91) | 0.136 | 1.90 (0.84-4.89) | 0.146 |  |  |  |  |
|  | Cluster 2 | 0.76 (0.29-2.12) | 0.586 | 0.77 (0.29-2.16) | 0.598 |  |  |  |  |
|  | Cluster 3 | 11.71 (5.71-28.26) | <0.001 | 10.38 (4.87-25.76) | <0.001 |  |  |  |  |
|  | Cluster 4 | 7.53 (3.64-18.26) | <0.001 | 6.79 (3.18-16.84) | <0.001 |  |  |  |  |
|  | Cluster 5 | 1.62 (0.71-4.18) | 0.278 | 1.57 (0.67-4.10) | 0.324 |  |  |  |  |
|  | Cluster 6 | 9.39 (3.96-24.83) | <0.001 | 9.01 (3.71-24.27) | <0.001 |  |  |  |  |
| Length of Stay (days) | Cluster 1 |  | <0.001 |  | <0.001 | 1.30 (1.17-1.44) | 1.23 (1.10-1.36) |  |  |
|  | Cluster 2 |  | <0.001 |  | <0.001 | 1.69 (1.52-1.87) | 1.58 (1.42-1.75) |  |  |
|  | Cluster 3 |  | <0.001 |  | <0.001 | 1.88 (1.69-2.09) | 1.61 (1.43-1.80) |  |  |
|  | Cluster 4 |  | <0.001 |  | <0.001 | 1.85 (1.67-2.06) | 1.62 (1.45-1.81) |  |  |
|  | Cluster 5 |  | <0.001 |  | 0.010 | 1.28 (1.15-1.42) | 1.15 (1.03-1.28) |  |  |
|  | Cluster 6 |  | <0.001 |  | <0.001 | 1.94 (1.66-2.27) | 1.75 (1.49-2.05) |  |  |
| Total hospital costs (CNY) | Cluster 1 |  | <0.001 |  | <0.001 |  |  | 1.61 (1.27-2.03) | 1.58 (1.27-1.96) |
|  | Cluster 2 |  | <0.001 |  | <0.001 |  |  | 1.86 (1.47-2.34) | 1.81 (1.45-2.24) |
|  | Cluster 3 |  | <0.001 |  | <0.001 |  |  | 4.02 (3.14-5.12) | 3.22 (2.55-4.06) |
|  | Cluster 4 |  | <0.001 |  | <0.001 |  |  | 2.88 (2.26-3.65) | 2.41 (1.91-3.02) |
|  | Cluster 5 |  | <0.001 |  | 0.003 |  |  | 1.49 (1.18-1.87) | 1.41 (1.13-1.75) |
|  | Cluster 6 |  | <0.001 |  | <0.001 |  |  | 3.58 (2.51-5.21) | 2.96 (2.12-4.19) |

**Table S3. Recommendation Counts From 20 Internal Validity Indices Across Candidate Cluster Solutions**

| **K** | **Number of indices** | **Indices recommending K** | **Joint highest count** |
| --- | --- | --- | --- |
| 2 | 3 | CH; McClain; Dunn | TRUE |
| 3 | 3 | Marriot; TraceW; Ball | TRUE |
| 4 | 1 | TrCovW | FALSE |
| 5 | 1 | Cindex | FALSE |
| 6 | 3 | Scott; Friedman; Ratkowsky | TRUE |
| 7 | 3 | KL; Hartigan; Rubin | TRUE |
| 8 | 2 | Silhouette; PtBiserial | FALSE |
| 9 | 1 | SDindex | FALSE |
| 10 | 3 | CCC; DB; SDbw | TRUE |

Candidate solutions ranged from K=2 to K=10. CH indicates Calinski-Harabasz; CCC, cubic clustering criterion; DB, Davies-Bouldin; KL, Krzanowski-Lai; and SD, scattering and distance.
